# Hepatitis C virus infection suppresses hepatitis B virus replication via the RIG-I-like helicase pathway

**DOI:** 10.1038/s41598-020-57603-9

**Published:** 2020-01-22

**Authors:** Kazuhiro Murai, Hayato Hikita, Yugo Kai, Yasuteru Kondo, Makoto Fukuoka, Keisuke Fukutomi, Akira Doi, Takuo Yamai, Tasuku Nakabori, Ryo Fukuda, Takeshi Takahashi, Kei Miyakawa, Hiroshi Suemizu, Akihide Ryo, Ryoko Yamada, Takahiro Kodama, Ryotaro Sakamori, Tomohide Tatsumi, Tetsuo Takehara

**Affiliations:** 1 Department of Gastroenterology and Hepatology, Osaka University Graduate School of Medicine, Osaka, Japan; 2 Department of Hepatology, Sendai Kousei Hospital, Sendai, Japan; 3 Laboratory Animal Research Department, Biomedical Research Laboratory, Central Institute for Experimental Animals, Kawasaki, Japan; 4 Department of Microbiology, Yokohama City University School of Medicine, Yokohama, Japan

**Keywords:** RIG-I-like receptors, Hepatitis B virus, Hepatitis C virus, Hepatitis B, Hepatitis C

## Abstract

Mechanisms of hepatitis B virus (HBV) reactivation after hepatitis C virus (HCV) elimination by direct-acting antiviral (DAA) treatment in HBV/HCV-co-infected patients remain unclear. We examined RIG-I-like helicase (RLH) pathway activation by HBV mono-infection, HCV mono-infection or HBV/HCV co-infection and interference between HBV and HCV in primary human hepatocytes. Interference between HBV and HCV and HBV reactivation after DAA treatment in humanized-liver mice were assessed. HCV infection activated RLH pathway, as evidenced by RIG-I, ISG15 and ISG56 expression induction; HBV caused only RIG-I induction *in vitro*. RLH activation was also found in HBV/HCV-co-infected cells, and HBV replication were suppressed in HBV/HCV-co-infected than in HBV-mono-infected cells. siRNA-mediated double knockdown of ISG15 and ISG56 increased HBV replication in HBV/HCV-co-infected cells. HCV infection activated RLH pathway and suppressed HBV replication in humanized-liver mice. Subsequent elimination of HCV by DAA administration downregulated RLH pathway and upregulated HBV replication in mice. RLH pathway was activated in livers of chronic hepatitis C patients compared to those of chronic hepatitis B or non-B, non-C patients. The RLH pathway activation was downregulated by HCV elimination. In conclusion, HCV infection activated RLH pathway and suppressed HBV replication in human hepatocytes. HCV elimination upregulated HBV replication, probably through RLH pathway downregulation.

## Introduction

The majority of chronic hepatitis C (CHC) patients are infected with hepatitis C virus (HCV) alone. However, in hepatitis B virus (HBV) endemic areas, there are patients who are co-infected with HBV and HCV^1–3^. The proportion of HCV antibody-positive patients in the worldwide population is approximately 1–4%. There are approximately 3.2–12.8 million individuals with HBV/HCV co-infection among 320 million HBV carriers^4^. HBV/HCV co-infection is frequently found in several high-risk populations, such as people who inject drugs (PWID)^5^. Clinical observations have shown that most HBV/HCV-co-infected patients have a lower titre of serum HBV DNA than HBV-mono-infected patients^6,7^. Nonetheless, it remains unclear whether interference between HBV and HCV exists in co-infected individuals.

In approximately 20–30% of hepatitis B surface (HBs) antigen-positive HBV/HCV-co-infected patients, treatment for HCV with direct-acting antivirals (DAAs) causes HBV reactivation^8–11^. Although some cases of HBV reactivation spontaneously remit^12^, HBV reactivation accompanied by hepatitis requiring anti-HBV therapy has been reported^13,14^. In general, anticancer drugs and immunosuppressants induce HBV reactivation^15^. Similarly, DAAs also cause HBV reactivation. How HBV increases after the successful treatment of HCV with DAAs remains unclear, as there is also no *in vitro* or *in vivo* model of HBV reactivation after the elimination of HCV by treatment with DAAs in basic research. Here, we reveal that HCV infection enhances the RIG-I-like helicase (RLH) system and suppresses HBV replication during HBV/HCV co-infection and that HCV elimination by DAA therapy decreases RLH system induction and enhances HBV replication.

## Results

### While HBV infection does not enhance the RLH system, HCV infection does enhance the RLH system

**Figure 1:**
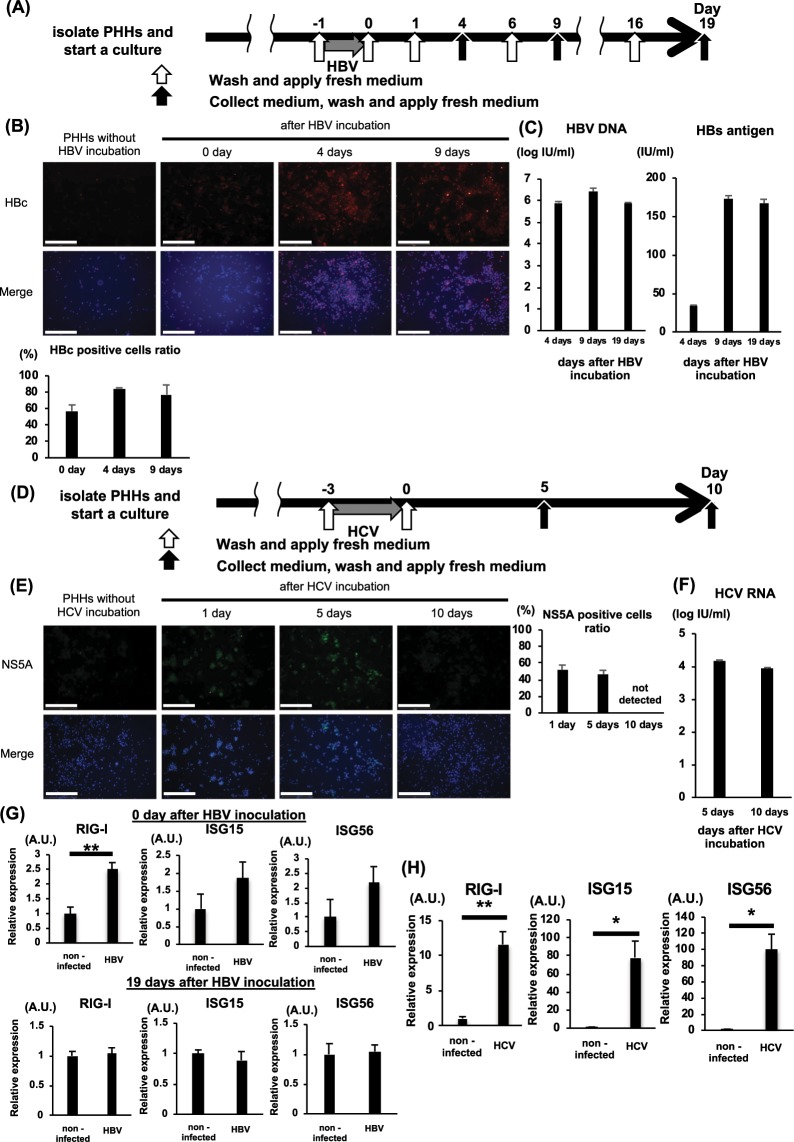
HBV or HCV infection of primary human hepatocytes from chimeric mice. Primary hepatocytes from chimeric mice were incubated with HBV (500 GEq/cell) for 1 day or with HCV (50 MOI) for 3 days. (**A**) Schematic of the HBV experimental procedure. (**B**) Representative pictures of immunofluorescent staining to determine HBc- and HBc-positive cell ratios on the indicated day (n = 4) The white bar in the pictures is a scale bar indicating 500 µm. (**C**) HBV DNA and HBs antigen in 3-day cultured medium (n = 4). (**D**) Schematic of the HCV experimental procedure. (**E**) Representative pictures of immunofluorescent staining to determine NS5A- and NS5A-positive cell ratios on the indicated day (n = 4). The white bar in the pictures is a scale bar indicating 500 µm. (**F**) HCV RNA in 5-day cultured medium (n = 3). (**G**) mRNA expression levels of RIG-1, ISG15 and ISG56 0 day and 19 days after HBV incubation (n = 4). (**H**) mRNA expression levels of RIG-1, ISG15 and ISG56 after HCV inoculation (n = 4). *p < 0.05, **p < 0.01, HBV-infected cells or HCV-infected cells vs. non-infected cells (PHHs infected with neither HBV nor HCV).

Primary human hepatocytes (PHHs) collected from humanized-liver chimeric TK-NOG mice (NOG mice expressing a thymidine kinase transgene) were incubated with HBV for 1 day (Fig. 1A). After HBV inoculation, 57.1% of hepatocytes were positive for HBV core (HBc) antigen by immunofluorescent staining. The HBc-positive cell ratio was approximately 80% at 4 days and 9 days after HBV incubation (Fig. 1B). HBs antigen and HBV DNA were detected in the supernatant of the PHHs at 4 days, 9 days and 19 days after HBV incubation (Fig. 1C). These data suggest that PHHs are susceptible to HBV, consistent with our previous report^16^. PHHs were incubated with HCV for 3 days (Fig. 1D). Then, HCV non-structural protein 5A (NS5A)-positive PHHs were detected 1 day and 5 days after HCV incubation, but not 10 days after HCV incubation, by immunofluorescent staining (Fig. 1E). The levels of HCV RNA were detected in the supernatant of the PHHs at both 5 days and 10 days after HCV incubation (Fig. 1F). To examine the influence of HBV and HCV on the RLH system, we investigated the expression levels of RIG-I and downstream ISG15 and ISG56 in PHHs. The mRNA levels of RIG-I were slightly higher in HBV-infected cells just after HBV inoculation than in non-infected cells, and no difference was found in ISG15 and ISG56 expression between the two groups of cells (Fig. 1G). No difference was found in RIG-I, ISG15 and ISG56 expression between HBV-infected cells 19 days after HBV inoculation and non-infected cells (Fig. 1G). In sharp contrast, the mRNA expression levels of RIG-I, ISG15 and ISG56 were drastically increased in HCV-infected cells compared with non-infected cells (Fig. 1H).

### HCV infection suppresses HBV replication and enhances the RLH system during HBV infection

**Figure 2:**
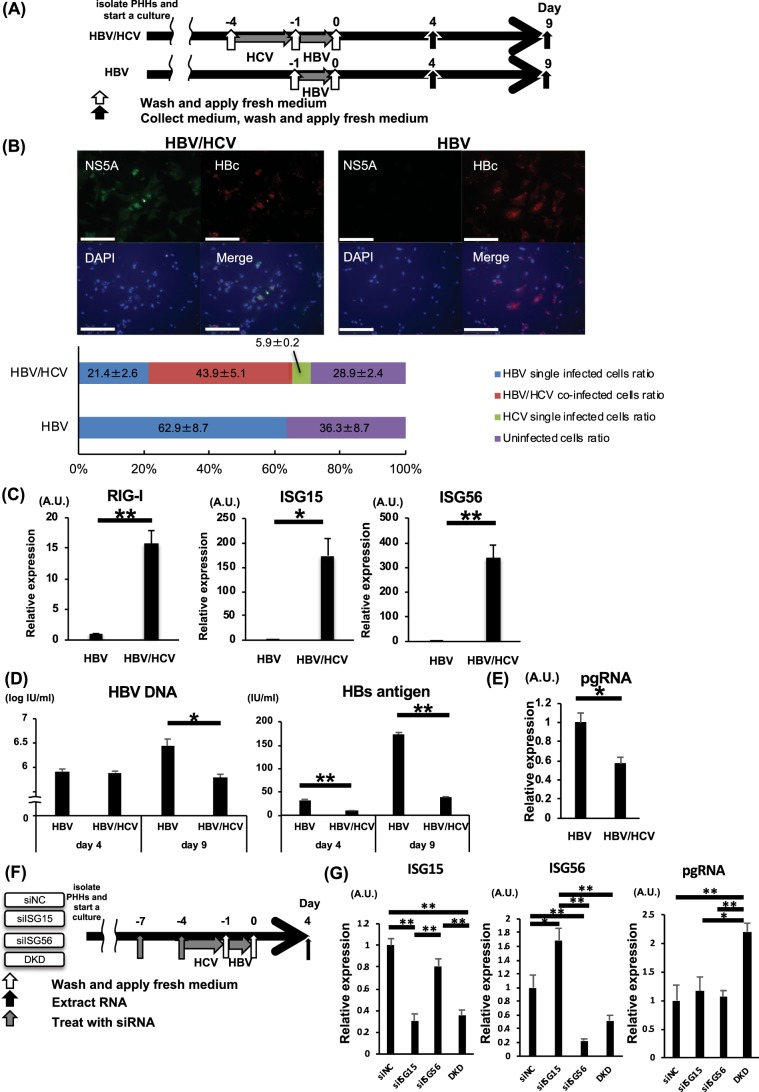
Co-infection of primary hepatocytes from chimeric mice with HBV and HCV. (**A**–**E**) PHHs were incubated with HCV (50 MOI) for 3 days, followed by incubation with HBV (500 GEq/cell) for 1 day to develop HBV/HCV-co-infected PHHs. (**A**) Schematic of the experimental procedure. (**B**) Representative pictures of immunofluorescent staining for HBc and NS5A after HBV incubation, HBc- and NS5A-positive cell ratios in HBV/HCV-co-infected PHHs and HBV-infected PHHs after HBV incubation. The white bar in the pictures is a scale bar indicating 200 µm. (**C**) mRNA expression levels of RIG-I, ISG15, and ISG56 4 days after HBV incubation (n = 4). (**D**) HBV DNA and HBs antigen levels in the supernatant 4 days and 9 days after HBV incubation (n = 4). (**E**) pgRNA levels at 4 days after HBV incubation (n = 4). F-G. PHHs were treated with siISG15 and/or siISG56 twice at 3 days before inoculated with HCV and at the same time of HCV inoculation. PHHs were incubated with HCV for 3 days, followed by incubation with HBV for 1 day. (**F**) Schematic of the experimental procedure with siRNA. (**G**) ISG15, ISG56 and pgRNA levels at 4 days after HBV incubation (n = 4). *p < 0.05, **p < 0.01.

The influence of HCV infection on HBV replication in PHHs was investigated. PHHs were incubated with HBV with or without prior HCV infection (Fig. 2A). Upon HBV infection, approximately two-thirds of the hepatocytes with or without prior HCV infection were positive for HBc. Approximately one-half of the HBV/HCV-co-infected cells were positive for NS5A, and importantly, cells positive for both HBc and NS5A were identified, indicating that co-infection in cultured human hepatocytes truly occurred (Fig. 2B). mRNA expression levels of RIG-I, ISG15 and ISG56 in PHHs after HBV incubation with prior HCV infection were significantly higher than those in PHHs without prior HCV infection (Fig. 2C). HBV DNA and HBs antigen levels in the supernatant of PHHs (Fig. 2D) and pre-genomic RNA (pgRNA) levels in PHHs (Fig. 2E) after HBV incubation with prior HCV infection were significantly lower than those in PHHs without prior HCV infection. To examine the impact of ISG15 and ISG56 increase in HCV-infected PHHs on HBV replication, ISG15- and/or ISG56-knocked down PHHs were incubated with HBV with prior HCV infection. Although the expression levels of pgRNA in ISG15-knocked down PHHs or ISG56-knocked down PHHs did not differ from those in control PHHs, pgRNA levels in ISG15 and ISG56 double-knocked down PHHs were significantly higher than control PHHs or single-knocked down PHHs (Fig. 2F,G).

### HCV infection inhibits HBV replication in chimeric mice infected with HBV and HCV

**Figure 3:**
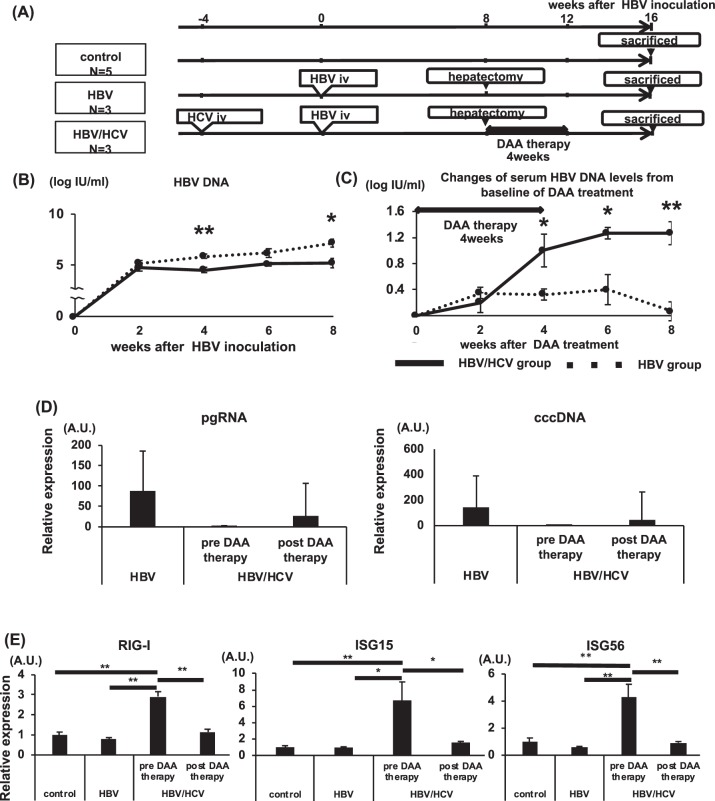
Co-infection of chimeric mice with HBV and HCV and elimination of HCV by treatment with DAAs. To develop HBV/HCV-co-infected mice, three chimeric mice were infected with HCV and then infected with HBV. To develop HBV-infected mice, three chimeric mice were infected with HBV. The HBV/HCV-co-infected mice were orally administered DAAs once a day for 4 weeks. Five chimeric mice without HBV or HCV infection in the control group were sacrificed. (**A**) Schematic of the experimental procedure. (**B**) Serum HBV DNA levels in HBV/HCV-co-infected mice and HBV-infected mice (n = 3). (**C**) Changes in serum HBV DNA levels from the baseline to when hepatectomy was performed (n = 3). (**D**) pgRNA levels and cccDNA levels in hepatocytes 8 weeks after HBV inoculation in HBV-infected mice and 8 weeks (pre-DAA therapy) and 16 weeks (post-DAA therapy) after HBV inoculation in HBV/HCV-co-infected mice (n = 3). (**E**) mRNA expression levels of RIG-I, ISG15 and ISG56 in the hepatocytes (n = 5: control, n = 3: HBV, pre-DAA therapy, post-DAA therapy). (B, C) *p < 0.05, **p < 0.01, HBV-infected mice vs. HBV/HCV-co-infected mice. (**E**) *p < 0.05, **p < 0.01.

To develop HBV/HCV-co-infected mice, three chimeric TK-NOG mice were infected with HCV and then infected with HBV. To develop HBV-infected mice, three chimeric mice were infected with HBV alone (Fig. 3A). The HBV/HCV-co-infected mice showed significantly lower serum HBV DNA levels than the HBV-infected mice (Fig. 3B). The HBV/HCV-co-infected mice were treated with DAAs for 4 weeks. HCV RNA was not detected in any HBV/HCV-co-infected mice from 2 weeks after the start of DAA therapy to sacrifice. The serum HBV DNA levels in the HBV/HCV-co-infected mice significantly increased after HCV elimination with DAAs (Fig. 3C). The pgRNA and cccDNA levels in the liver tissues of the HBV/HCV-co-infected mice were lower than those of the HBV-infected mice, and pgRNA and cccDNA levels increased after the elimination of HCV with DAAs (Fig. 3D). The mRNA levels of RIG-I, ISG15 and ISG56 in the hepatocytes of the HBV/HCV-co-infected mice before treatment were higher than those of mice infected with the HBV-infected mice, and the RIG-I, ISG15 and ISG56 mRNA levels decreased after HCV elimination with DAAs (Fig. 3E).

### The RLH pathway was activated in HCV-infected livers of CHC patients

**Figure 4:**
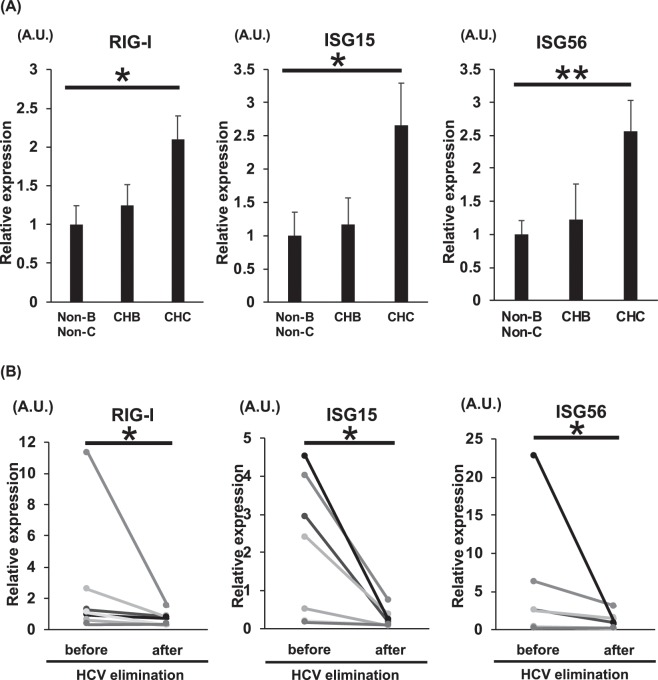
RLH pathway in livers of CHB, CHC, and non-B, non-C patients. A. Sixty-five liver biopsy samples were obtained from CHB, CHC and non-B, non-C patients. (**A**) mRNA levels of RIG-I, ISG15 and ISG56 in their livers. B. Seven paired liver biopsy samples before and after HCV elimination by DAA treatments were obtained from CHC patients with DAA treatments. (**B**) mRNA levels of RIG-I, ISG15 and ISG56 in their livers. *p < 0.05, **p < 0.01.

The mRNA expression levels of RIG-I, ISG15 and ISG56 in human liver biopsy samples from CHB, CHC, and non-B, non-C patients were measured by real-time reverse transcription PCR (RT-PCR) (Table 1). In human liver biopsy samples from CHC patients, the mRNA expression levels of RIG-I, ISG15 and ISG56 were higher than those in liver biopsy samples from CHB patients and non-B, non-C patients (Fig. 4A). We further examined using pared liver biopsy samples from CHC patients before and after HCV elimination by treatment (Table 2). Liver biopsy samples before HCV elimination were obtained at the start of treatment with DAAs. Liver biopsy samples after HCV elimination were obtained 48 weeks after the end of treatment (EOT) with DAAs. RIG-I, ISG15 and ISG56 levels were decreased by HCV elimination (Fig. 4B).

**Table 1:** Characteristics of the human liver samples.

	non-B, non-C	CHB	CHC
N	**17**	11	37
Sex (male/female)	0/17	8/3	18/19
Median age (years) (range)	62 (24–74)	45 (25–78)	65 (19–79)
Median AST (IU/L) (range)	101 (17–767)	44 (28–163)	43 (5–182)
Median ALT (IU/L) (range)	104 (18–714)	56 (19–467)	40 (10–292)
Median HBV DNA (log IU/ml) (range)	—	6.0 (2.2 − >8.2)	—
Median HCV RNA (log IU/ml) (range)	—	—	6.2 (4.1–7.2)

**Table 2:** Characteristics of the human liver samples obtained from CHC patients before and after HCV elimination.

	before HCV elimination	after HCV elimination
N	7	
Sex (male/female)	4/3	
Median age (years) (range)	75 (53–81)	
Median HCV RNA (log IU/mL) (range)	5.8 (2.4–6.6)	not detected
Median Platelets (×10^4^/μL) (range)	18.2 (6.6–25.6)	16.2 (8.1–28.3)
Median PT (%) (range)	93.9 (69.2–118.7)	97.4 (67.6–114.4)
Median AST (IU/L) (range)	55 (14–91)	14 (8–34)
Median ALT (IU/L) (range)	37 (14–123)	21 (13–41)
Median Total bilirubin (mg/dL) (range)	0.97 (0.68–1.89)	0.93 (0.65–2.31)
Median Albumin (g/dL) (range)	4.3 (3.9–4.4)	4.2 (4–4.8)

## Discussion

Although patients are sometimes co-infected with HBV and HCV, the interaction between HBV and HCV infection has been rarely examined due to the lack of a suitable co-infection system. There has been no report examining the interaction between HBV and HCV infection in normal hepatocytes. In the present study, we revealed that HBV and HCV could co-infect PHHs and addressed the interaction between HBV and HCV using these PHHs. This study is the first to examine the impact of HCV infection on HBV replication levels in normal hepatocytes *in vitro* and *in vivo*.

It has been reported that RIG-I and melanoma differentiation-associated gene-5 (MDA-5) in the cytoplasm and endosome are involved in the recognition of HCV RNA and general viral RNA^17^. RIG-I has also been reported to recognize HCV RNA in the early phase of infection and activate downstream host innate immunity^18^. In the present study, the mRNA levels of RIG-I, ISG15, and ISG56 increased during HCV infection *in vitro* and in mouse experiments. They are also increased in HCV-infected livers of CHC patients. Interferon-stimulated genes (ISGs) have broad antiviral activity. ISG15 is a critical ISG with antiviral activity against DNA and RNA viruses^19^. ISG56 limits HBV replication by regulating posttranscriptional steps^20^. In the present study, suppression of both ISG15 and ISG56, but not suppression of each alone, increased pgRNA levels, suggesting that ISG15 and ISG56 collaboratively suppress pgRNA levels. It is thought that in patients with HBV/HCV co-infection, HBV replication is strongly suppressed by HCV-induced upregulation of ISGs, including ISG15 and ISG56. Consequently, HCV elimination with DAAs decreases ISGs expression leading to enhancement of HBV replication.

HBV reactivation is caused by anticancer drugs or immunosuppressants^15^. Anticancer drugs in the clinic that cause HBV reactivation mainly fall into two classes: traditional cytotoxic chemotherapy drugs that mainly suppress T cells and biological agents related to B cells such as rituximab^21^ or obinutuzumab^22^. Most immunosuppressants, such as cyclosporine A^23^ or tacrolimus^23^, mainly suppress T cells. Unlike anticancer drugs, DAAs do not have cytotoxic effects on B cells or T cells. Although they also do not have immunosuppressive effects, DAAs do cause HBV reactivation. In addition, in the present study, treatment with DAAs resulted in HBV reactivation in immunodeficient TK-NOG mice, indicating that HBV reactivation proceeded without immune cells.

In conclusion, HCV infection suppressed HBV replication. At the same time, HCV enhanced the RLH pathway. In chimeric TK-NOG mice, elimination of HCV reversed HBV suppression. At the same time, elimination of HCV also reversed the enhancement of the RLH pathway. These data suggest that the enhancement of the RLH pathway induced by HCV infection during HBV/HCV co-infection contributes to inhibition of HBV replication. The reversal of RLH pathway enhancement by the elimination of HCV using DAAs contributes to HBV reactivation in HBV/HCV-co-infected patients after treatment with DAAs.

## Materials and Methods

### Cell culture

PHHs were collected from chimeric mice with a chimeric liver rates of 50% or more using the two-step collagenase-pronase liver perfusion method in the same manner as previously reported^24^. All experiments began within 10 days of collecting PHHs from chimeric mice. The PHHs were seeded on 12- or 24-well collagen type I-coated microplates (AGC TECHNO GLASS CO., LTD., Shizuoka, Japan). Culture medium containing 2% dimethyl sulfoxide (DMSO) (NACALAI TESQUE, INC., Kyoto, Japan) was changed on the indicated days.

The culture supernatant of HepG2.2.15 cells was used as HBV inoculum *in vitro*. HepG2.2.15 cells have an integrated HBV genome, and HBV genotype D is present in the culture supernatant^25^. The culture supernatant of HepG2.2.15 cells was collected every 3 days, filtered through a 0.45-μm filter (Merck Millipore, Burlington, MA, USA), concentrated 200 times, and used as the inoculum. PHHs were incubated with culture medium containing the HBV inoculum and 4% polyethylene glycol 8000 (Promega, Fitchburg, WI, USA) for 24 hours. After incubation with the HBV inoculum, the PHHs were washed three times with phosphate-buffered salts (PBS) containing 2% DMSO, and the culture medium was changed.

The culture supernatant of Huh7 cells inoculated with JFH-1, which belongs to HCV genotype 2a^26^, was used as the HCV inoculum *in vitro*. Culture supernatant from Huh7 cells inoculated with JFH-1 was collected every 3 days, filtered through a 0.45-μm filter, and used as the inoculum. PHHs were incubated with culture medium containing the HCV inoculum for 72 hours, after which the PHHs were washed three times with PBS containing 2% DMSO, and the culture medium was changed.

### Humanized-liver chimeric TK-NOG mice

Humanized-liver chimeric TK-NOG mice were prepared as previously described^27^. The human hepatocyte chimeric rate is correlated with serum human albumin levels, and the estimated human hepatocyte chimeric rate can be calculated from serum human albumin levels^27^. Serum human albumin levels were measured with a Human Albumin ELISA Quantitation Set (Bethyl Laboratories Inc., Montgomery, TX, USA). All mouse experiments were conducted with the Guide for the Care and Use of Laboratory Animals from Osaka University Graduate School of Medicine and Central Institute for Experimental Animals (CIEA).

### HBV or HCV inoculation of mice

CHB patient serum (genotype C, 8.5 log IU/ml) and CHC patient serum (genotype C, 6.8 log IU/ml) were used for mouse experiments under the approval of the Institutional Review Board for Clinical Research at Osaka University Hospital (12050, 13048). Written informed consent was obtained from these patients for experimental use of their sera. The CHB patient serum used in the experiment was diluted to 7.5 log IU/ml. One hundred μl of diluted CHB patient serum or CHC patient serum was injected intravenously into chimeric TK-NOG mice with a chimeric rate of 50% or more as previously described^28^. Mouse blood samples were collected from the external jugular vein. The protocols involving animal experiments were approved by the Animal Care and Use Committee of Osaka University Medical School.

### Co-infection of chimeric TK-NOG mice with HBV and HCV and elimination of HCV in HBV/HCV-inoculated chimeric mice by treatment with DAAs

To develop HBV/HCV-co-infected mice, three humanized-liver chimeric TK-NOG mice were infected with HBV 4 weeks after HCV infection. To develop HBV-infected mice, three humanized-liver chimeric TK-NOG mice were infected with HBV. The HBV/HCV-co-infected mice were orally administered with DAAs (40 mg/kg asunaprevir +30 mg/kg daclatasvir +20 mg/kg beclabuvir) once a day for 4 weeks. Asunaprevir, daclatasvir, and beclabuvir were provided by Bristol-Myers Squibb (New York, NY, USA). Eight weeks after HBV infection, hepatectomy was performed in HBV-infected mice and HBV/HCV-co-infected mice to collect liver samples. Sixteen weeks after HBV inoculation, the chimeric HBV-infected mice and HBV/HCV-co-infected mice were sacrificed. Five humanized-liver chimeric mice without HBV or HCV infection were sacrificed as control mice.

### Liver biopsy samples

Liver biopsy samples were obtained from CHB, CHC and non-B, non-C patients with approval by the Institutional Review Board for Clinical Research at Osaka University Hospital (15267). Liver biopsy samples were obtained from patients before and after HCV elimination by DAA therapy with approval by the Institutional Review Board for Clinical Research at Sendai Kousei Hospital (30–12). Informed consent was obtained in writing from these patients for experimental use of their liver biopsy samples. Residual liver biopsy samples were used for the experiment.

### Measurement of HBV DNA and HBs antigen levels and HCV RNA levels

HBV DNA levels were measured using a COBAS TaqMan HBV Test (Roche Diagnostics, Rotkreuz, Switzerland). HBs antigen levels were measured using a chemiluminescence enzyme immunoassay (CLEIA System, Fujirebio, Tokyo, Japan). HCV RNA levels were measured using a COBAS TaqMan HCV Test (Roche Diagnostics, Rotkreuz, Switzerland). The samples were diluted 10-fold (for *in vitro* experiments) or 100-fold (for *in vivo* experiments) for measurement.

### RNA extraction and RT-PCR

Total RNA was extracted using an RNeasy Mini Kit (Qiagen, Venlo, Netherlands). The extracted RNA was treated with DNase (RNase-Free DNase Set, Qiagen, Venlo, Netherlands) to analyse pgRNA. To synthesize cDNA, the extracted RNA was reverse transcribed with Rever Tra Ace qPCR RT Master Mix (Toyobo, Tokyo, Japan). For RT-PCR, the following primers in TaqMan gene expression assays were used: human β-actin (Hs99999903_m1, Thermo Fisher Scientific, Waltham, MA, USA), human RIG-I (Hs01061436_m1, Thermo Fisher Scientific, Waltham, MA, USA), human IRF3 (Hs01547283_m1, Thermo Fisher Scientific, Waltham, MA, USA), human ISG15 (Hs01921425_s1, Thermo Fisher Scientific, Waltham, MA, USA), and human ISG56 (Hs03027069_s1, Thermo Fisher Scientific, Waltham, MA, USA). To analyse pgRNA, the following forward and reverse primer set was prepared: 5′-TGTCCTACTGTTCAAGCCTCCAA-3′ (forward) and 5′-GAGAGTAACTCCACAGTAGCTCCAA-3′ (reverse). Human β-actin was used as the internal control, and all mRNA expression levels were normalized to the levels of the internal control mRNA.

### DNA extraction and real-time PCR for HBV cccDNA

Total DNA was extracted using with the QIAamp DNA Mini Kit (Qiagen, Venlo, Netherlands) according to the manufacturer’s protocol. To detect Genotype C cccDNA, the following primer set and probe were used: primer set, 5′-TCCCCGTCTGTGCCTTCTC-3′ (1420–1438) and 5′-GCACAGCTTGGAGGCTTGA-3′ (1738–1756); probe, 5′-FAM-CCGTGTGCACTTCG-3′ (1449–1462). PCR was performed at 50 °C for 2 minutes, 94 °C for 10 minutes, and 45 cycles of 94 °C for 30 seconds and 60 °C for 90 seconds. HBV cccDNA levels were normalized to the quantified expression levels of human RNase P using the TaqMan RNase P Control Reagents Kit (#4316844, Thermo Fisher Scientific, Waltham, MA, USA).

### Transfection of siRNA against ISG15 and ISG56

Two siRNAs against human ISG15(s18524) and ISG56 (s7151), and the negative controls (#4390843) were purchased from Thermo Fisher Scientific (Waltham, MA, USA). Transfections were performed using Lipofectamine RNAiMAX (Thermo Fisher Scientific, Waltham, MA, USA) according to the manufacturer’s protocol.

### Immunofluorescent staining

Cells were grown on chambered coverglasses (Matsunami Glass Ind., Ltd., Osaka, Japan), washed with PBS, and then fixed with ice-cold acetone-methanol. Cells were washed again (3× PBS) and blocked in PBS/0.2% BSA. HBc antigen monoclonal antibodies were generated by immunizing of BALB/c mice with recombinant HBc protein synthesized in a wheat cell-free protein production system, as previously described^29^. HBc antigen was probed by using a mouse monoclonal antibody against HBc antigen (clone 7B2, culture supernatant of the hybridoma)^30^ diluted 100-fold in PBS (1 hour at room temperature). HCV NS5A was stained by using a rabbit monoclonal antibody against NS5A diluted 100-fold in PBS (1 hour at room temperature). As a secondary antibody, goat anti-mouse antibody labelled with Alexa Fluor 594 (Cell Signaling Technology, Danvers, MA, USA) or goat anti-rabbit antibody labelled with Alexa Fluor 488 (Cell Signaling Technology, Danvers, MA, USA) diluted 500-fold in PBS was used. Cell nuclei were stained using DAPI. Stained cells were analysed using a fluorescence microscope (Invitrogen EVOS FL Auto 2 Imaging System; Thermo Fisher Scientific, Waltham, MA, USA).

### Statistical analysis

The data are presented as the means ± standard error. Comparisons between two groups in the *in vitro* studies were performed by an unpaired two-sided t test. Comparisons between two groups in the *in vivo* studies were performed by an unpaired two-sided t test. For the *in vitro*, *in vivo* and liver biopsy samples from the patients of chronic liver disease, analysis of variance (ANOVA) was performed to detect an overall difference among multiple groups, followed by the Tukey-Kramer test. Comparisons between before and after HCV elimination in the paired liver biopsy samples were performed by Wilcoxon signed-rank test. A value of P < 0.05 was used to indicate statistical significance.
